# Benefits of Distinguishing between Physical and Social-Verbal Aspects of Behavior: An Example of Generalized Anxiety

**DOI:** 10.3389/fpsyg.2016.00338

**Published:** 2016-03-14

**Authors:** Irina Trofimova, William Sulis

**Affiliations:** Collective Intelligence Laboratory, Department of Psychiatry and Behavioral Neurosciences, McMaster UniversityHamilton, ON, Canada

**Keywords:** GAD, temperament, FET model, sociability, sex differences

## Abstract

Temperament traits and mental illness have been linked to varying degrees of imbalances in neurotransmitter systems of behavior regulation. If a temperament model has been carefully structured to reflect weak imbalances within systems of behavior regulation, then in the presence of mental illness, these profiles should exhibit distinct patterns consistent with symptoms of mental illness. In contrast to other temperament models used in studies of anxiety disorders, the Functional Ensemble of Temperament (FET) model differentiates not only between emotionality traits, but also between traits related to physical, social-verbal and mental aspects of behavior. This paper analyzed the predictions of the FET model, which maps 12 functional aspects of behavior to symptoms of generalized anxiety disorder (GAD) as described in the DSM/ICD. As an example, the paper describes a study of the coupling of sex, age and temperament traits with GAD using the FET framework. The intake records of 116 clients in treatment with confirmed diagnosis of GAD in a private psychological practice were compared using ANOVA against records of 146 healthy clients using their scores on the FET-based questionnaire, in age groups 17–24, 25–45, 46–65. Patients with GAD in all age groups reported significantly lower Social Endurance, Social Tempo, Probabilistic reasoning (but not in physical aspects of behavior) and higher Neuroticism than healthy individuals, however, no effects on the scales of Motor Endurance or Tempo were found. These findings show the benefits of differentiation between motor-physical and social-verbal aspects of behavior in psychological assessment of mental disorders.

## Functional Perspective and Functional Ensemble of Temperament Model (FET) as a Framework for Mapping Individual Differences

The classification of individual differences in psychology has developed from several different perspectives. The Five-Factors model, for example, was developed based on a factor analysis of lexical descriptors of personality characteristics, overlooking a strong sociability bias among these descriptors. Language developed historically to optimize people’s socialization, consequently, language has more words describing aspects of socialization in comparison to those describing physical and mental aspects of behavior. Language also reflects a negativity bias of emotionality, with more words describing negative than positive emotions (see Trofimova, 2014; Trofimova and Sulis, submitted for details). Thus studies using the Five Factor model consistently show large Extraversion and Neuroticism factors – reflecting the sociability and negative emotionality biases in language rather than the deeper structure of individual differences.

One alternative approach to human diversity is to map the systems of behavior regulation in terms of the functional aspects of behavioral tasks. Systems of behavior regulation developed in evolution in tune with aspects of human activities that were universal across tasks (i.e., their duration, changeability, social vs. physical nature, level of abstraction, etc). Analyzing systems from the perspective of a small set of such universal functional features of activities opens the possibility for a more compact and formal presentation of human diversity, including symptoms of mental disorders. This functional perspective was implemented in the neurochemical “Functional Ensemble of Temperament (FET)” model that was developed utilizing the Structure of Temperament Questionnaire (Rusalov and Trofimova, 2007; Trofimova, 2010a,b; Trofimova, 2015b; Trofimova and Robbins, 2016).

Temperament, i.e., biologically based individual differences in healthy people, and mental illnesses are considered as varying degrees along the same continuum of neurotransmitter imbalance in neurophysiological systems of behavioral regulation (Clark et al., 1994; Heath et al., 1994; Mehrabian, 1995; Ball et al., 1999; Weinstock and Whisman, 2006; Brown, 2007; Rusalov and Trofimova, 2007; Weiss et al., 2009; Karam et al., 2010; Trofimova, 2015b). Many temperament traits (such as impulsivity, sensation seeking, neuroticism, endurance, plasticity, sociability, extraversion) have been linked to brain neurotransmitters and hormonal systems, i.e., the very same systems implicated in mental disorders (Gray, 1982; Cloninger, 1986; Heath et al., 1994; Kagan et al., 1994; Depue and Morrone-Strupinsky, 2005; Trofimova and Sulis, 2010; Zentner and Shiner, 2012; Trofimova, 2015b; Trofimova and Robbins, 2016; Trofimova and Sulis, submitted).

A temperament model, carefully structured to reflect weak imbalances within neurotransmitter systems of behavior regulation (emerging as temperament), should exhibit distinct profiles in the presence of illness consistent with DSM-V symptoms of such illness. The majority of studies investigating the coupling of temperament with mental illness describe associations of emotionality-related traits of temperament but have poor differentiation between non-emotionality aspects of behavior. Thus, anxiety disorders were associated with higher scores on Neuroticism/Negative Affect scales within Watson’s Positive/Negative Affects model (Clark et al., 1994; Weinstock and Whisman, 2006; Brown, 2007; Sellbom et al., 2008), Mehrabian’s model (Mehrabian, 1995), Cloninger’s model (TCI) (Heath et al., 1994), Trofimova’s FET model (Trofimova and Sulis, 2010; Trofimova and Christiansen, 2016; Trofimova and Sulis, submitted), Akiskal’s model (Karam et al., 2010) and the Big Five model (Ball et al., 1999; Weiss et al., 2009; Kotov et al., 2010; Watson and Naragon-Gainey, 2014). Neuroticism, however, appeared to be high in many types of mental illness and therefore did not differentiate between mental disorders. For example, in addition to the association between high Neuroticism and anxiety disorders, Neuroticism/Negative Affect was also reported to have significant positive correlation with depression (Mineka et al., 1998; Ball et al., 1999; Weinstock and Whisman, 2006; Brown, 2007; Sellbom et al., 2008; Karam et al., 2010; Kotov et al., 2010; Watson and Naragon-Gainey, 2014; Trofimova and Christiansen, 2016). Patients with histrionic personality disorder and major depression also scored higher on the Harm Avoidance scale (similar to Neuroticism, it describes an anxious disposition to novelty and uncertainty) in studies using Cloninger’s TCI test (Cloninger, 1986; Kusunoki et al., 2001; Farmer and Seeley, 2009). This global coupling of the trait of Neuroticism with mental disorder shows that the scales measuring Neuroticism/Negative Affects in various temperament models are not sufficient to differentiate between different aspects of healthy behavior and between types of diagnoses in mental illness.

In contrast, the FET model considers 12 temperament traits in a 3 × 4 matrix: nine activity-related traits (energetic, dynamic, and orientational) each assessed in three domains (physical, social, and intellectual) together with three systems related to emotionality (Neuroticism, Impulsivity, and Self-confidence). For example, energetic systems emerge in temperament as traits of Endurance, i.e., the ability of an individual to sustain prolonged and/or intense activities. The FET model considers three types of Endurance – Physical, Social, and Intellectual (mental, i.e., attention). Differentiation between physical and social aspects of behavior in psychological assessment and in models of human diversity was first proposed by Nebylitzyn and Rusalov within the longest experimental tradition in studying properties of nervous systems (Strelau, 1998; Rusalov and Trofimova, 2007). Using human subjects, they performed a wide range of psychophysical experiments such as evoked potentials, absolute thresholds in visual, auditory, and tactile modalities, strengths of excitation and mobility in auditory and visual modalities. This research resulted in the first activity-specific model of temperament linking temperament traits to the functional components (aspects) of activities and also separating physical and social traits of temperament (Rusalov, 1979, 1989; Rusalov and Trofimova, 2007).

Rusalov’s model was recently revised (Rusalov and Trofimova, 2007; Trofimova, 2010a,b; Trofimova and Sulis, 2011) and complemented by an analysis of the functionality of neurochemical systems in the form of the FET. The nine FET non-emotionality traits are posited to be regulated by monoamine (MA) and neuropeptide systems, whereas the three emotionality-related traits emerge from a dysregulation of opioid receptors systems that have direct control over MA systems (see Trofimova, 2015b; Trofimova and Robbins, 2016 for details). The FET model suggests that there is no one-to-one correspondence between the neurotransmitter systems underlying temperament traits (or mental disorders) but instead specific ensemble associations between these systems emerge as temperament traits.

For example, FET views the Neuroticism trait as a slight disregulation within kappa- (KOPr), mu-opioid receptors (MOPrs) systems and their regulation of MA release, which in more extreme dysregulation can lead to GAD. This suggestion is based on the findings that kappa-opioid receptors (KOPr) are important players in chronic anxiety, behavioral mobilization and arousal, and in animals with KOPr deficiency the expression of stress-related hormones is significantly reduced (degli Uberti et al., 1995; Yamauchi et al., 1997; Carlezon et al., 2009; Schwarzer, 2009; Bruchas et al., 2010; Bodnar, 2011). A dysregulation of MOPr receptors (normally suppressing KOPr activation but failing to do so in chronic anxiety) brings not only behavioral arousal but also a component of dysphoria, resulting from imbalances in the supply/demand of endorphins binding these receptors. Since both KOPr and MOPr regulate release of MAs, this can explain the links between MAs and anxiety disorders. There is a solid body of evidence that KOPr activation induces noradrenalin release and the HPA axis arousal (Takahashi et al., 1990; Simonin et al., 1995; Filliol et al., 2000; Tanaka et al., 2000; Phelps et al., 2006; Reyes et al., 2007, 2009; Schwarzer, 2009; Wittmann et al., 2009). Both KOPr and MOPr systems appear to regulate not only noradrenalin release but also GABA release (also linked to anxiety disorders) and reciprocal glutamate release (Bodnar, 2011) in a selective manner (Tanaka et al., 2000; Nagi and Piñeyro, 2011).

There are neurochemical systems that are specifically linked to social aspects of behavior, such as prolactin (Panksepp, 1998) and oxytocin (Bielsky and Young, 2004; Depue and Morrone-Strupinsky, 2005; Taylor et al., 2006; Donaldson and Young, 2008; Barraza and Zak, 2009). Both serotonin and MOPrs systems regulate oxytocin and vasopressin, and are also linked to social behavior (Keverne and Curley, 2004; Barr et al., 2008; Way et al., 2009). The interaction between MOPr, serotonin, noradrenalin and oxytocin systems suggests that GAD might be marked by a decrease in social (affiliative) aspects of behavior, when MOPr and MA systems are out of balance.

For many years it was reported that temperament traits were stable or static over time, unchanging after age 30. However, many age-related changes are linked to hormonal, endocrine and neuropeptidic changes, and since temperament traits are also based on neurochemical systems of behavior regulation, it is reasonable to suggest that there might be age differences in correlations between temperament traits and the scales measuring mental illness, especially physical, and social. Similarly, sex differences, also linked to hormonal and endocrine systems, might emerge as differences in temperament profiles and susceptibility to mental illness. For example, it is well-documented that men have on average greater upper body strength, higher rates of risk- and sensation seeking and openness to experience (Costa et al., 2001; Zuckerman, 2007; Trofimova, 2015a). Women, on the other hand, have higher rates of sociability in comparison to men (Trofimova, 2013, 2015a).

### Generalized Anxiety Disorder: An Example

Here we present an example of the benefits obtained from differentiating between physical, social-verbal, and mental aspects of behavioras proposed by the FET model. The hypothesis of this study was in line with the main DSM-V descriptor of GAD related to the presence of *worrying*: this symptom was expected to emerge in higher scores on the Neuroticism scale in GAD patients, in comparison to controls. In addition, the FET hypothesis predicted that GAD patients might have a decrease in Social-verbal Endurance (sociability). Assessment data from the intake records of 262 (M/F = 111/151) Canadians, patients and associates of a private psychiatric and psychological practice (Psychological Services 4018 having four distant locations in Southern Ontario, Canada) were analyzed. The practice has its own Late Life Memory Clinic (Haldimand War Memorial Hospital, Dunnville, ON, Canada) that provides screening for dementia in clients and patients over 60. The sample was divided into those who were diagnosed with GAD (*N* = 116, M/F = 47/69) and those who did not have mental illness (*N* = 146, M/F = 64/82). GAD patients were divided into three age groups: Age1: 17–24 years-old (*Mean age* 19.42, *SD* = 2.13), M/F = 21/31, *N* = 52; Age2: 25–45 years-old (*M* = 35.28, *SD* = 6.13), M/F = 14/20, *N* = 34, Age3: 46–65 years-old (*M* = 53.86, *SD* = 5.45), M/F = 12/18, *N* = 30. Subsample of healthy subjects was also divided into age groups of the same range: Age1: *N* = 70, M/F = 28/42; Age2: *N* = 46, M/F = 23/23; Age3: *N* = 30, M/F = 13/17. The prevalence of females in the sample reflected the female prevalence in patients with GAD in the general population. The subsample of healthy people was structured to reflect the structure of the GAD subsample to enable a balanced statistical analysis. The “Anxiety” group consisted of patients diagnosed with generalized anxiety disorder (GAD) (moderate to severe) on the basis of the structured DSM-IV clinical interview, file review, testing and a prolonged period of subsequent treatment. Testing included the Beck Anxiety Inventory (GAD group included patients with scores of 36 or higher), State Trait Anxiety Inventory (scores of 61 or higher), Post-Traumatic Stress diagnostic scale and Symptom CheckList-90 (scores of 31 or higher on the Anxiety scale).

#### Procedure and Measures

All healthy participants of this study, as well as non-dementing clients and patients gave consent allowing the use of their intake forms for research purposes. During either intake testing (for patients and clients) or research (for healthy participants) each person completed the Compact Structure of Temperament Questionnaire (STQ-77) (Rusalov and Trofimova, 2007; Trofimova, 2010a,b; Trofimova and Sulis, 2011)^1^. The STQ-77 consists of 77 statements, assigned to 12 temperament scales (six items each) and a validity scale (five items, addressing social desirability bias), which are listed below. Subjects responded according to a 4-point Likert scale format: (1) “strongly disagree,” (2) “disagree,” (3) “agree,” (4) “strongly agree.”

The temperament scales are organized in groups as following:

1–3: Endurance scales – Motor (ERM, alpha Cronbach for this data = 0.81), Social (ERS, α = 0.78) and Intellectual Endurance (ERI, α = 0.73) (the ability of an individual to sustain prolonged physical, social or mental activity, respectively).
4–5: Dynamic scales – Motor (TMM, α = 0.80) and Social Tempo (TMS, α = 0.74) (preferred speed of physical activity, or speed of speech and reading and of other verbal activities, respectively) and Plasticity (PL, α = 0.72), (the ability to adapt quickly to changing situations, to change plans, to shift between tasks).
6–9: Orientation scales – Sensation Seeking (SS, α = 0.73), (the degree of orientation of a person to basic physical sensations and pleasures; tendency for sensation-seeking and risk-taking behavior); Empathy (EMP, α = 0.74), (orientation to other people’s emotional states), Sensitivity to Probabilities (PRO, α = 0.72), (orientation to probabilistic expectations, causes, and consequences of events, the efficient extraction and processing of new knowledge).
10–12: Emotionality scales – Self-confidence (SLF, α = 0.71), (the tendency to be optimistic and confident in one’s own performance, to ignore warnings and criticism); Impulsivity (IMP, α = 0.73), (speed of emotionally driven actions (emotional reactivity), poor ability to control immediate impulses for actions); Neuroticism (NEU, α = 0.77), (low tolerance of uncertainty and novelty, negativity bias in expectations of outcomes in own activity).
13: Validity scale – social desirability tendency in answers. Results within the range of 15–20 on the validity scale should be considered invalid as the respondents are likely to demonstrate positive impression bias in their responses.

*Statistical processing* included descriptive scale statistics (Means, Standard Deviations, alpha Cronbach coefficient for STQ scales). The mean scores on the STQ scales from the groups contrasted by Diagnosis (GAD vs. Controls), Age (three groups) and Sex were submitted to multi-factorial ANOVA to examine the impact these three factors. *Post hoc* comparisons were performed using both the Tukey and Fisher LCD tests with an alpha level of 0.05. The partial Eta-squared values (η^2^) were also calculated as an additional metric of effect size for all significant ANOVA contrasts.

## Differential Contribution of Physical-Motor and Social-Verbal Traits in Gad, Age and Sex Differences

**FIGURE 1:**
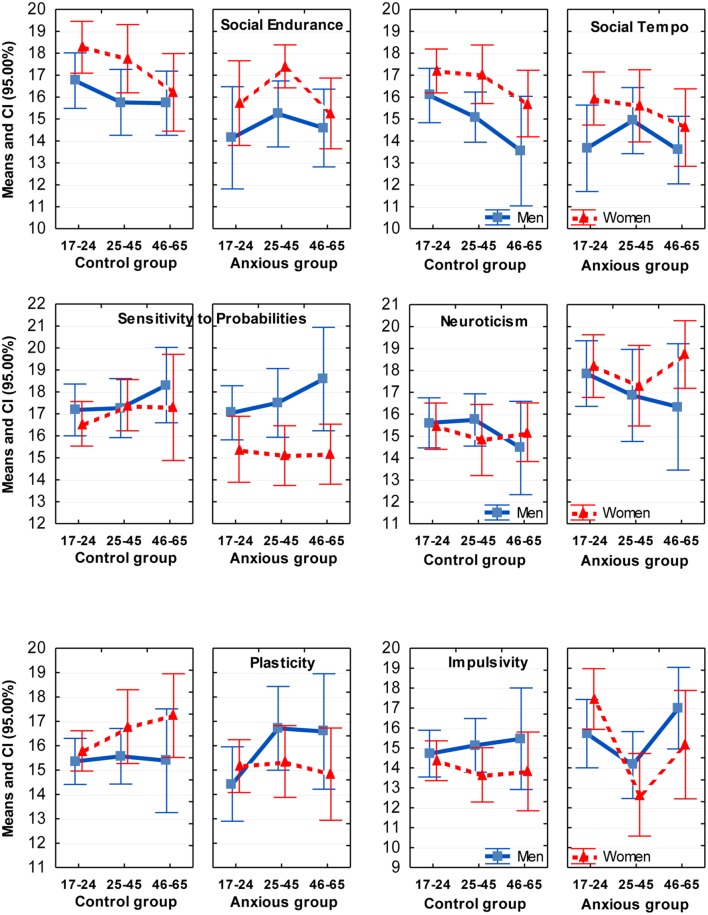
**Means and Confidence Intervals (CI) of the scores on temperament scales which showed coupling with GAD (first four graphs).** Sex differences were especially significant on the scales of Social Tempo and Sensitivity to Probabilities. Interaction effects with GAD with sex were found on the scales of Plasticity and Sensitivity to Probabilities; with age – on the scale of Impulsivity.

The comparison of GAD and control groups showed that the presence of GAD was associated with significant effects on four traits. Anxious patients reported significantly lower Social Endurance, lower Social Tempo, lower Sensitivity to Probabilities and higher Neuroticism, in comparison to the control group (**Table 1**; **Figure 1**). The finding of significantly higher Neuroticism in GAD patients, in comparison to non-anxious participants is consistent with reports using other temperament models to study mental illness, with a scale of Neuroticism as part of those models as cited above. More importantly, Social Endurance (sociability) and Social Tempo were reported as decreased in the presence of GAD but Motor Endurance or Motor Tempo were unaffected. This selectivity in the effect of GAD on the energetic and dynamical aspects of activity suggests that the DSM-V criterion of fatigue as a symptom of GAD is more nuanced, and that this is observable utilizing an activity specific model such as the FET. Low sociability can interfere with a patients’ ability to sustain an effective support network and to seek out help and therefore might not be a visible aspect of GAD to the community.

**Table 1:** Means and Standard Deviations (*M_SD_*) on the STQ-77 scales for groups contrasted by GAD (all ages combined), ANOVA effects for Anxiety factor and η^2^ for Anxiety, Sex, and Age factors.

STQ-77	Controls *N* = 146	Anxious *N* = 116	Effect of	Anxiety	Sex	Age
Scales	*M_SD_*	*M_SD_*	*F*(1,250)	η^2^	η^2^	η^2^
Motor Endurance	16.87_3.77_	16.48_3.41_	1.18	0.005	0.022*	0.018
Motor Tempo	16.78_3.32_	16.27_3.23_	0.21	0.001	0.012	0.027*
Sensation Seeking	15.50_3.63_	14.73_3.94_	1.39	0.005	0.147	0.150
Social Endurance	17.64_3.66_	15.84_4.00_	11.92***	0.045	0.021*	0.002
Social Tempo	16.12_3.31_	14.90_3.49_	5.90*	0.23	0.048***	0.028*
Empathy	16.85_3.49_	16.89_4.09_	0.01	0.001	0.007	0.001
Intellectual Endurance	16.48_3.10_	15.87_3.67_	0.67	0.002	0.004	0.021
Plasticity	15.96_2.95_	15.34_3.31_	1.55	0.006	0.001	0.019
Sensitivity to Probabilities	17.16_3.26_	16.19_3.43_	4.03*	0.046	0.046***	0.009
Self-Confidence	16.12_3.04_	15.28_3.68_	2.19	0.009	0.000	0.005
Impulsivity	14.47_3.32_	15.51_4.42_	2.88	0.011	0.012	0.038**
Neuroticism	15.31_3.18_	17.71_3.72_	26.52***	0.095	0.004	0.007

*p < 0.05, **p < 0.01, ***p < 0.001; *N* = 292.

The differentiation between 12 traits within the FET model also appeared to be beneficial in assessment of age differences. Three significant effects of age were found in this study: lower scores on Motor Tempo and Social Tempo were reported by subjects in the older age groups, i.e., dynamical traits but not endurance- or orientation-related related traits. Such decrease is consistent with reports that speed of activities declines with age in both men and women (Birren and Schaie, 1990; Trofimova and Christiansen, 2016; Trofimova and Sulis, submitted). A significant interaction effect between age and GAD appeared in relation to Impulsivity (*F* = 4.00, *p* = 0.019; η^2^ = 0.031). In the presence of GAD the youngest (17–24 years-old) women reported higher Impulsivity than other groups whereas in groups aged 25–65 women appeared to have the best impulse control, even in the GAD group.

There were four significant sex differences: Motor Endurance and Sensitivity to Probabilities were reported higher by men, while Social Endurance and Social Tempo were reported higher by women, consistent with other reports using young age samples (Bishop et al., 1987; Nyborg, 1994; Collaer and Hines, 1995; Trofimova, 2013). There were three significant interactions between Sex and Anxiety factors, on the scales of Sensitivity to Probabilities (*F* = 5.30, *p* = 0.022; η^2^ = 0.021), Plasticity (*F* = 5.80, *p* = 0.016, η^2^ = 0.023) and Self-confidence (*F* = 4.55, *p* = 0.033, η^2^ = 0.018). *Post hoc* analysis showed that anxious women reported significantly lower scores on these scales, in comparison to the anxious men and to non-anxious women. Interestingly, a rather long held cultural stereotype that portrays women as being more neurotic and histrionic than men was most definitely *not* supported in this study. Healthy men and women across all age groups reported similar scores in Neuroticism and Self-confidence. Sex differences emerged only in the presence of GAD. It appeared that the presence of GAD was associated with lower scores on Self-confidence, Plasticity and Sensitivity to Probabilities in women much more than in men.

## Conclusion

The FET framework for differentiating between 3 emotionality aspects and nine functional aspects of behavior: endurance, speed of integration of an action and orientation, considered separately in physical, social, and mental domains, appears to be quite useful for studying individual differences. This framework offers dimensions for mapping symptoms of mental illness, such as GAD. Such dimensionality can bring more insights about symptoms of mental illness, sex and age differences. For example, our study found that the current GAD criterion of fatigue would benefit from being related more to social-verbal and less to physical aspects of endurance. Future directions include studies investigating the benefits of differentiating between 12 components of the FET model in samples of patients with various mental illnesses. Examples of potential applications of this perspective relate to: (1) a new classification of psychiatric disorders based on functional aspects of arousal described within the FET model, as a contribution to the NIH initiative on Research Domain Criteria (Insel, 2014; Trofimova and Sulis, submitted); (2) new insights for research in psychopharmacology, and (3) mapping temperamental profiles associated with dispositions to specific psychiatric disorders.

## Author Contributions

WS participated in the development of the study, in data collection, and analysis, in writing and editing the manuscript and in giving final approval. IT led in the development of the study, in data collection and analysis, in writing and editing the manuscript and in giving final approval.

## Conflict of Interest Statement

The authors declare that the research was conducted in the absence of any commercial or financial relationships that could be construed as a potential conflict of interest.

## Footnotes

1 The complete validation history can be found in Rusalov and Trofimova (2007) and Trofimova (2010a,b).
